# Three new species of *Synodontella* (Monogenea, Ancyrocephalidae), gill parasites of *Synodontis* spp. (Siluriformes, Mochokidae) from Côte d’Ivoire

**DOI:** 10.1051/parasite/2019044

**Published:** 2019-07-25

**Authors:** Enoutchy F. Bouah, Valentin N’Douba, Antoine Pariselle

**Affiliations:** 1 Laboratory of Hydrobiology, UFR Biosciences, Felix Houphouët Boigny University 22 BP 582 Abidjan 22 Côte d’Ivoire; 2 ISEM, Univ. Montpellier, CNRS, IRD, CC 065 Place Eugène Bataillon 34095 Montpellier Cedex 5 France; 3 Laboratory “Biodiversity, Ecology and Genome”, Research Center “Plant and Microbial Biotechnology, Biodiversity and Environment”, Mohammed V University in Rabat, Faculty of Sciences 4 avenue Ibn Batouta B.P. 1014 Rabat Morocco

**Keywords:** Siluriformes, Mochokidae, *Synodontis*, Monogenean, *Synodontella*, Gill

## Abstract

Four species of *Synodontella*, including three new, are reported from three species of *Synodontis* (*S. ocellifer*, *S. nigrita*, and *S. velifer*) from north-western Côte d’Ivoire. *Synodontella melanoptera* Dossou & Euzet, 1993 was found on the gills of *S. nigrita* and *S. velifer* and was already known from the gills of *S. melanopterus* in Benin and the gills of *S. obesus* and *S. rebeli* in Cameroon. The new species are *Synodontella speroadotevii* n. sp. from *S. nigrita* (type-host), *S. velifer* and S. *ocellifer*, and *Synodontella bagoueensis* n. sp. and *Synodontella akengboi* n. sp., both from *S. velifer* (type-host for both), *S. nigrita* and *S. ocellifer*. The new species differ from the other species of *Synodontella* mainly in the morphology of their male copulatory organs (MCO): *Synodontella speroadotevii* has a two-part penis (one being a hollow tube and the other a flattened tube); *Synodontella bagoueensis* has a wide G-shaped penis with a sub-terminal opening; and *Synodontella akengboi* has a simple narrow J-shaped penis. *Synodontella speroadotevii* differs from *Synodontella bagoueensis* and *Synodontella akengboi* in the shape of the dorsal transverse bar, which shows no protuberance, and also in the morphology of the MCO. *Synodontella bagoueensis* differs from the other two species in that it has a dorsal transverse bar that is V-shaped and a G-shaped MCO. *Synodontella akengboi* differs from the two other species in having a J-shaped MCO and in the size of its ventral and dorsal anchors which, contrary to the other two species, are almost similar.

## Abstract (fr)

Quatre espèces de *Synodontella*, dont trois nouvelles, sont signalées chez trois espèces de *Synodontis* (*S. ocellifer*, *S. nigrita* et *S. velifer*) du nord-ouest de la Côte d’Ivoire. *Synodontella melanoptera* Dossou & Euzet, 1993 a été trouvé sur les branchies de *S. nigrita* et *S. velifer* et était déjà connu des branchies de *S. melanopterus* au Bénin et des branchies de *S. obesus* et *S. rebeli* au Cameroun. Les nouvelles espèces sont *Synodontella speroadotevii* n. sp. de *S. nigrita* (hôte type), *S. velifer* et *S. ocellifer* et *Synodontella bagoueensis* n. sp. et *Synodontella akengboi* n. sp., tous deux de *S. velifer* (hôte type pour les deux), *S. nigrita* et S. *ocellifer*. Les nouvelles espèces se distinguent des autres espèces de *Synodontella* principalement par la morphologie de leurs organes copulateurs mâles (OCM) : *Synodontella speroadotevii* a un pénis en deux parties (l’une étant un tube creux et l’autre un tube aplati) ; *Synodontella bagoueensis* a un large pénis en forme de G avec une ouverture subterminale ; et *Sy. akengboi* a un pénis simple et étroit en forme de J. *Synodontella speroadotevii* diffère de *Sy. bagoueensis* et *Sy. akengboi* par la forme de la barre transversale dorsale, qui ne montre aucune protubérance, ainsi que dans la morphologie de l’OCM. *Synodontella bagoueensis* se distingue des deux autres espèces par sa barre transversale dorsale en forme de V et un OCM en forme de G. *Synodontella akengboi* diffère des deux autres espèces par son OCM en forme de J et par la taille de ses crochets ventraux et dorsaux qui, contrairement aux deux autres espèces, sont presque similaires.

## Introduction

Monogeneans that infect small catfishes of Mochokidae have been the focus of several studies [2, 3, 6, 7, 10, 13] and, in Africa, only 10 species of *Synodontella* Dossou & Euzet, 1993 (hereafter *Sy*.) have thus far been reported from catfishes belonging to *Synodontis* Cuvier (hereafter *S.*)

In 1968, Paperna and Thurston [10] described *Sy. synodontii* (Paperna & Thurston, 1968), the type species, from the gills of *S. victoriae* Boulenger in Uganda. In 1993, Dossou and Euzet [2] reported *Sy. acropenis* Dossou & Euzet, 1993 from the gills of *S. sorex* Günther, *Sy. melanoptera* Dossou & Euzet, 1993 from the gills of *S. melanopterus* Boulenger, and *Sy. davidii* Dossou & Euzet, 1993 from the gills of *S. membranaceus* (Geoffroy Saint-Hilaire) from Benin and Mali. In 1995, Douëllou and Chishawa [3] described *Sy. zambezensis* Douëllou & Chishawa, 1995 from *S. zambezensis* Peters from Lake Kariba in Zimbabwe. Raphahlelo et al. [13] confirmed the validity of these five species in their revision of the latter and Mbondo et al. [7] more recently redescribed *Sy. melanoptera* from the gills of *S. obesus* Boulenger and *S. rebeli* Holly in the Sanaga River (Cameroon). Mbondo et al. [7] also described two new species: *Sy. apertipenis* Mbondo, Nack & Pariselle, 2017 and *Sy. sanagaensis* Mbondo, Nack & Pariselle, 2017 from the gills of *S. rebeli*. Furthermore, Mbondo et al. [6] in 2019 reported *Sy. angustupenis* Mbondo, Nack & Pariselle, 2019 from the gills of *S. nummifer* Boulenger, and *Sy. longipenis*, Mbondo, Nack & Pariselle, 2019, and *Sy. simplex* Mbondo Nack & Pariselle, 2019 from the gills of *S. decorus* Boulenger from the Boumba River (Cameroon).

The study presented herein on the gill monogeneans of mochokid *Synodontis* catfishes is the first carried out in Côte d’Ivoire. The fish, all caught from the Bagoué River, were *S. nigrita* Valenciennes, *S. velifer* Norman, and *S. ocellifer* Boulenger. We found four species of monogeneans belonging to *Synodontella.* One of them, *Sy. melanoptera*, was already known from the gills of *S. melanopterus*, *S. obesus*, and *S. rebeli* [2, 7], whereas the other three represent species new to science and are described here.

## Materials and methods

**Figure 1:**
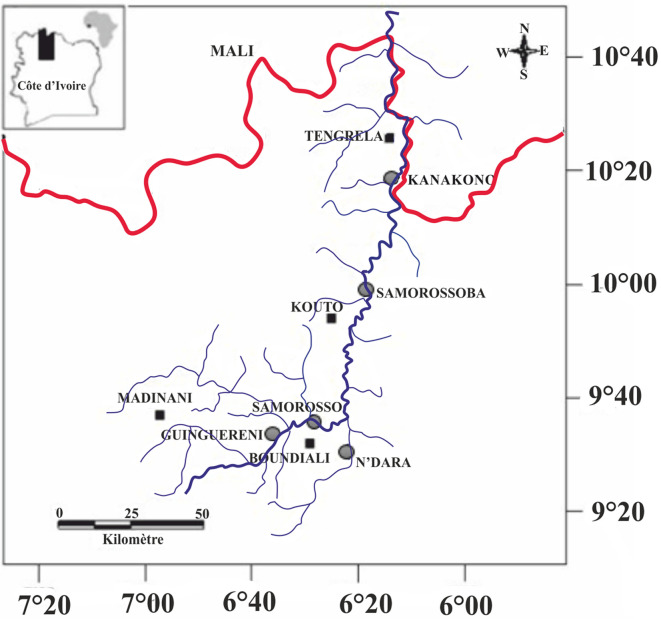
Locations of the different sampling stations on the Bagoué River, Côte d’Ivoire.

**Figure 2:**
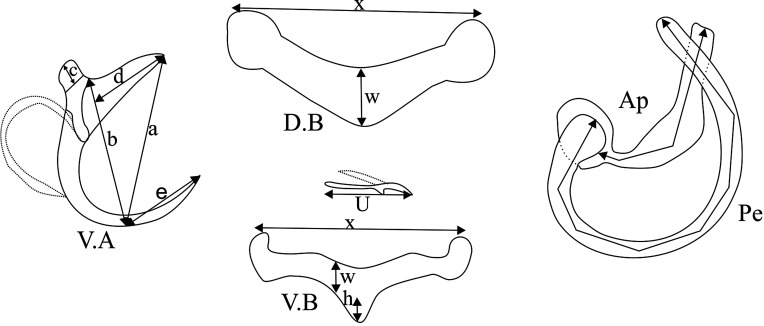
Measures used in this study. V.A: ventral anchor; *a*: total length; *b*: blade length; *c*: shaft length; *d*: guard length; *e*: point length. D.B: dorsal bar; *w*: maximum width; the arrow for length (x) of Ventral Bar has no extremities. V.B, ventral transverse bar; *h*, length of protuberance; *w*, maximum width; *x*, length. Ap: accessory piece length. Pe: penis length. *u*, length of marginal hooks.

Examined fish (Table 1) were caught with gillnets in the Bagoué River (north-western Côte d’Ivoire) from August 2018 to January 2019 at the following localities: Kanakono (10°18′N, 6°13′W); Samorossoba (6°21′W; 9°52′N); Samorosso (6°30′W; 9°34′N); N’Dara (6°24′W; 9°26′N) and Guinguereni (6°35′W; 9°32′N) (Fig. 1). Fish were identified on site upon capture using the key developed by Paugy et al. [12] and their gills resected into two sections, one ventral and one dorsal, and fixed and stored in liquid nitrogen. Upon return to the laboratory, gill arches were thawed and intensely rinsed to detach the monogeneans, which were individually collected and transferred directly onto a slide in a drop of glycerin ammonium-picrate mixture (GAP) [5]. Each specimen was covered with a coverslip and, after complete diffusion of the mounting medium, sealed with Glyceel. A microscope (Motic BA310) with an integrated camera was used for observations. Identification of monogeneans was based on the morphology and size of the sclerotized pieces of the haptor and the copulatory complex. Measurements were made as shown in Figure 2 following Dossou and Euzet [2]. All measurements (average followed in parentheses by minimum − maximum) are in micrometers (μm). Types were deposited in the Muséum National d’Histoire Naturelle, Paris, France (MNHN) and the Royal Museum for Central Africa, Tervuren, Belgium (RMCA).

**Table 1:** The four species of *Synodontella* (*Sy*.) encountered on the three species of *Synodontis* (*S*.) examined from the River Bagoué, Côte d’Ivoire. Infection parameters given as number of parasites per host species followed by prevalence and mean intensity in parentheses; host standard length given as mean followed by min−max, in cm.

Host	*S. nigrita*	*S. velifer*	*S. ocellifer*
Standard length	30 (6.5–11)	15 (9.5–17)	10 (9.8–14)
Parasite	Infection parameters		
*Sy. speroadotevii*	9 (30%–0.3)	10 (66.7%–1.4)	6 (60%–0.9)
*Sy. bagoueensis*	30 (100%–11.6)	14 (91.7%–23.6)	5 (57%–1.4)
*Sy. akengboi*	25 (80%–4.7)	12 (75%–7.2)	7 (70%–8.6)
*Sy. melanoptera*	15 (50%–6.4)	5 (33.3%–2.8)	0

## Results

The four monogenean species found on the fish examined (Table 1) comply with the description of members of *Synodontella* given by Dossou and Euzet [2] in having the following characteristics: Ancyrocephalidae; presence of three pairs of cephalic glands; ocellae present or absent. Intestinal branches united posteriorly. Haptor armed with two pairs of anchors (one dorsal and one ventral), two central bars (one dorsal, one ventral), and 14 hooks (seven pairs). Ventral hooks articulated at the lateral ends of the ventral bar and characterized by a handle traversed by a hull leading to the blade guard limit. Testis median and posterior. Vas deferens surrounding the left intestinal branch. Presence of a seminal vesicle and a globular prostatic reservoir. Simple tubular penis with accessory piece. Pre-testicular median ovary. Lateral vitelline cells. Right lateral vaginal opening. Muscular vagina not sclerotized. Presence of a seminal receptacle. Gill parasites of African Mochokidae (Siluriformes).

Three of these species are new to science and are described below. *Synodontella melanoptera* Dossou & Euzet, 1993, was already known from *S. melanopterus* in Benin and *S. rebeli* and *S. obesus* in Cameroon; it is herein reported for the first time from Côte d’Ivoire from *S. nigrita* and *S. velifer.*

## *Synodontella speroadotevii* n. sp.

urn:lsid:zoobank.org:act:B59860CC-ECF6-404D-945C-4D1540AFB7BC

Type Host: *Synodontis nigrita* Valenciennes, 1840.

Other hosts: *Synodontis velifer* Norman and *Synodontis ocellifer* Boulenger.

Infection site: Gills.

Type locality: Bagoué River, Samorossoba, Côte d’Ivoire (9°52′N; 6°21′W).

Other localities; Kanakono (6°13′W; 10°18′N); Samorosso (6°30′W; 9°34′N); N′Dara (6°24′W; 9°26′N) and Guinguereni (6°35′W; 9°32′N).

Type specimens: Holotype MNHN HEL909, paratypes MNHN HEL910-911.

Etymology: The species epithet *“speroadotevii”* honours Mr. Stanislas Spero-Adotevi, who has worked extensively for UNICEF in Africa.

### Description

**Figure 3:**
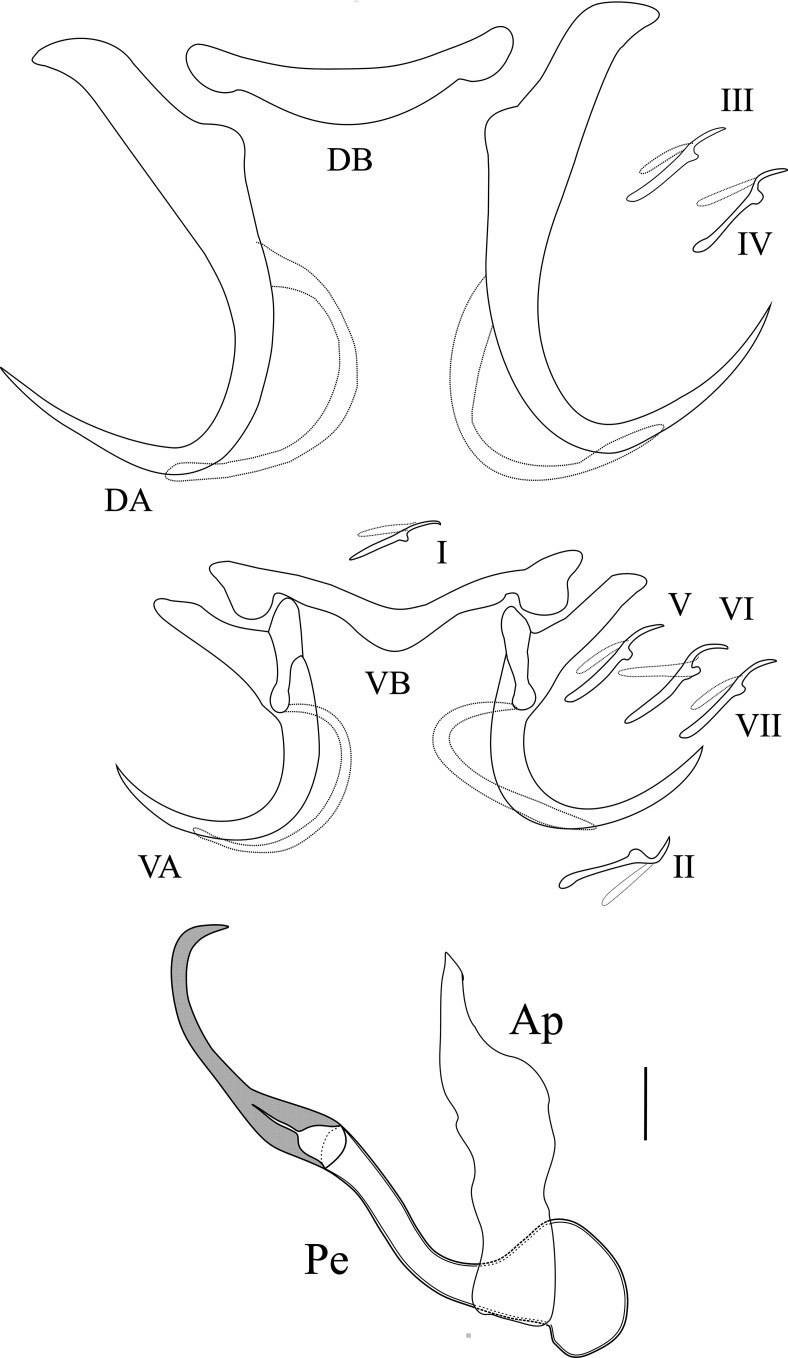
Hard parts of *Synodontella speroadotevii* n. sp. D.A: dorsal anchor; D.B: dorsal transverse bar; V.A: ventral anchor; V.B: ventral transverse bar; I–VII: marginal hooks; Ap: accessory piece; Pe: penis. Scale bar = 10 μm.

Based on 25 individuals (Fig. 3). Dorsal anchors, large, with guard longer than wide and characterized by an extremely short shaft: *a* = 58.4 (52–63), *c* = 3.1 (2–4), *d* = 21.3 (18–25). Long arched blade terminated by a long point: blade *b* = 46.3 (42–50), *e* = 29 (24–34). Dorsal transverse bar, simple and slightly curved, with swollen ends: *x* = 40.5 (38–43), *w* = 7.7 (6–8). Ventral anchors significantly smaller than dorsal ones, with long guard and a short shaft traversed by a hull finished by a small button in the concavity of the blade, presence of a filament at the end of the hull that covers the blade, which is strongly arched, with a long point: *a* = 34.2 (31–36), *b* = 26.9 (25–29), *c* = 3.6 (2–6), *d* = 15.4 (11–18), *e* = 21.1 (16–23). Long ventral transverse bar *x* = 47 (40–51) with notches at its ends but no median protuberance. All marginal hooks are small and have retained their larval morphology. The penis Pe = 88.1 (81–96), with ovoid bulb at its base, is in two parts of equal length. The first one is a simple, wide and slightly S-shaped tube with the opening at its extremity, the second one is a curved extension of the penis wall, highly sclerotized and not hollowed. Accessory piece, attached to basal ovoid bulb, is short, simple, wide and straight Ap = 34, 7 (30–41). No sclerotized vagina.

### Remarks

*Synodontella speroadotevii* n. sp. differs from all its congeneric species mainly in the morphology of the penis, with its opening being in the middle of its course, which is unique amongst *Synodontella* spp. It also differs in its ventral transverse bar, which shows no median protuberance.

## *Synodontella bagoueensis* n. sp.

urn:lsid:zoobank.org:act:C65C6C1F-0CF0-4EE0-9F99-A280ADDC905D

Type host: *Synodontis velifer* Norman.

Other hosts: *Synodontis nigrita* Valenciennes and *Synodontis ocellifer* Boulenger.

Infection site: Gills.

Type locality: Bagoué River, Samorosso, Côte d′Ivoire (9°34′N; 6°30′W).

Other localities: Kanakono (6°13′W; 10°18′N); Samorossoba (6°21′W; 9°52′N); N′Dara (6°24′W; 9°26′N) and Guinguereni (6°35′W; 9°32′N).

Type specimens: Holotype MNHN HEL912, paratypes MNHN HEL913-914.

Etymology: “*bagoueensis*” refers to the type locality, Bagoué River.

### Description

**Figure 4:**
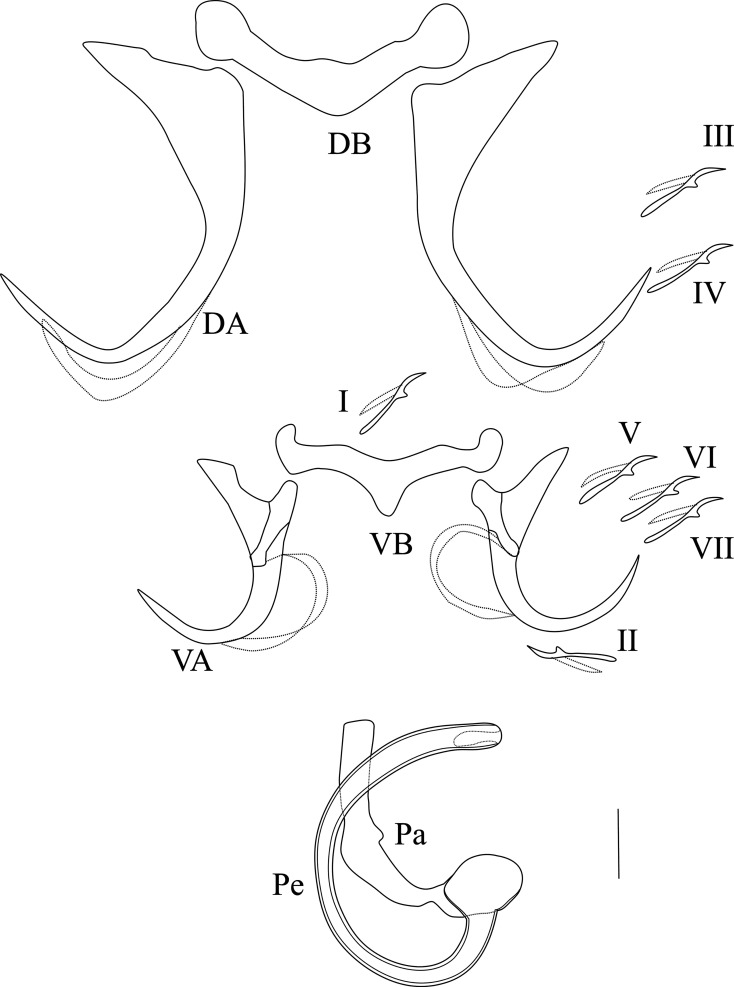
Hard parts of *Synodontella bagoueensis:* D.A: dorsal anchor; D.B: dorsal transverse bar; V.A: ventral anchor; V.B: ventral transverse bar; I–VII: marginal hooks; Ap: accessory piece; Pe: penis. Scale bar = 10 μm.

Based on 25 individuals (Fig. 4): Large dorsal anchors *a* = 47.1 (43–53), with a long guard more than six times the shaft length: *c* = 3.2 (2–4). Guard long and large: *d* = 19.3 (16–22). Blade long: *b* = 45.3 (41–51) terminated by a long point: *e* = 20.1 (18–24). Dorsal transverse bar *x* = 37.6 (32–45) V-shaped, thick *w* = 8.2 (6–11), with swollen ends. Ventral anchors *a* = 26.7 (25–30) smaller than dorsal ones, with guard longer than shaft. Guard elongated, more than three times the shaft length *d* = 12.9 (9–15), *c* = 4.3 (3–6). Arched blade, ending by long point: *b* = 22 (20–24), *e* = 14.9 (13–26). Hull, with swollen extremities, ends in the concave part of the blade. Ventral transverse bar, *x* = 33 (28–37) long, *w* = 4.1 (2–6) wide, with slightly rounded ends and short median expansion *h* = 3.1 (2–5). All marginal hooks are small and have retained their larval morphology. The tubular penis Pe = 88.2 (67–92), consists of a wide G-shaped tube with constant diameter; the opening (hardly visible) seems to be sub-terminal. The accessory piece, Ap = 31.3 (21–38) simple, starts from the base of the penis. No sclerotized vagina.

### Remarks

*Synodontella bagoueensis* n. sp. is close to *Sy. apertipenis* in the morphology of the ventral transverse bar with rounded ends and having ventral anchors with a highly arched blade and a long guard. However, *Sy. bagoueensis* can be distinguished from *Sy. apertipenis* by: (1) the dorsal anchor with a long, wide guard and residual shaft *vs* short, thin guard and a marked shaft, (2) the dorsal transverse bar V-shaped, thick with rounded ends *vs* enlarged slightly split ends, and (3) the male copulatory organ a long tube G-shaped *vs* long curved tube widely open from its third part.

## *Synodontella akengboi* n. sp.

urn:lsid:zoobank.org:act:8AD2A638-7038-4EBD-B346-E4F8104ED52D

Type host: *Synodontis velifer* Norman.

Other hosts: *Synodontis nigrita* Valenciennes and *Synodontis ocellifer* Boulenger.

Infection site: Gills.

Type locality: Bagoué River, Samorosso, Côte d’Ivoire (6°30′W; 9°34′N).

Other localities: Kanakono (10°18′; 6°13′W); Samorossoba (9°52′N; 6 °21′W); N’Dara (6°24′W; 9°26′N) and Guinguereni (9°32′N; 6°35′W).

Type specimens: Holotype MNHN HEL915, paratypes MNHN HEL916-917.

Etymology: the epithet *akengboi* honours Professor Gilbert Marie Aké-Ngbo of Félix Houphouët-Boigny University.

### Description

**Figure 5:**
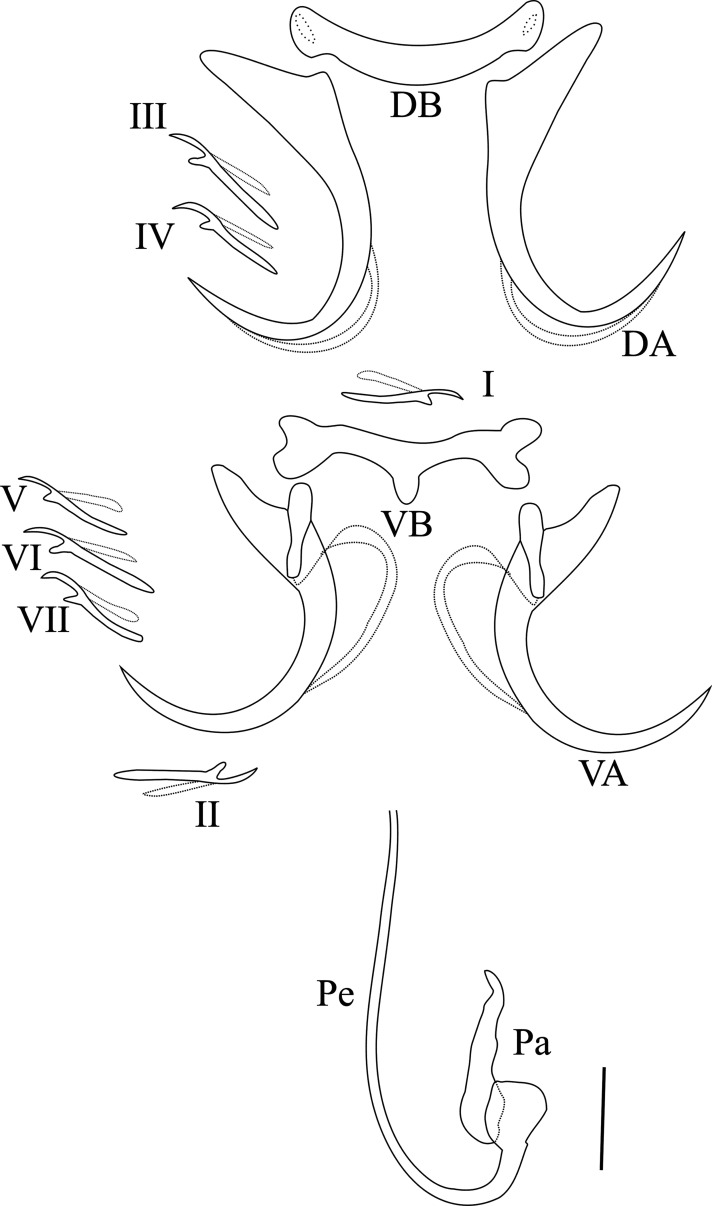
Hard parts of *Synodontella akengboi;* D.A: dorsal anchor; D.B: dorsal transverse bar; V.A: ventral anchor; V.B: ventral transverse bar; I–VII: marginal hooks; Ap: accessory piece; Pe: penis. Scale bar = 10 μm.

Based on 30 specimens (Fig. 5). Dorsal anchors *a* = 36.7 (33–39) with very short shaft and more pronounced guard, curved blade with long point *b* = 31.3 (28–34), *e* = 16.5 (11–19), *d* = 11.7 (9–15), *c* = 2.8 (2–4). Dorsal transverse bar *x* = 28.1 (25–31), simple, slightly curved with swollen ends, *w* = 4.4 (3–5). Ventral anchors, *a* = 32 (29–34) approximatively equal in size to the dorsal ones, with guard which is three times shaft length. Blade strongly arched with a long point, hull ended by a small button in the concavity of the blade, *b* = 27.3 (24–30), *c* = 4.1 (2–6), *d* = 11.8 (8–15), *e* = 17.4 (13–21). Ventral transverse bar *x* = 28.1 (25–33), with a notch at each extremity and a median expansion *w* = 3.4 (2–5), *h* = 4 (2–5). All the small marginal hooks have retained their larval morphology. The penis Pe = 81.6 (57–96), tubular, thin, J-shaped, has at its base a simple accessory piece Ap = 29.4 (18–50). No sclerotized vagina.

### Remarks

*Synodontella akengboi* n. sp. is similar to *Sy. zambezensis* in the morphology of the dorsal transverse bar, which is slightly curved, and by having a ventral transverse bar with notches at both ends and a median protuberance. However, *Sy. akengboi* is mainly distinguishable from *Sy. zambezensis* by its ventral anchors and via the morphology of the male copulatory organ. The blade of the ventral anchors of *Sy. akengboi* is strongly arched *vs* angled at a right angle in *Sy. zambezensis*. The male copulatory organ of *Sy. akengboi* is a long tube (81.6 μm) and is thin and J-shaped with a simple shorter accessory piece, while the male copulatory organ of *Sy. zambezensis* consists of a fan-shaped tube at its distal end and possesses a thin accessory piece.

## Discussion

In the present study, representatives of four species of monogeneans were collected from three host species. Three of these parasites are new to science. This finding brings to 13 the known total number of species of these monogeneans belonging to *Synodontella* described from the siluriform Mochokidae in Africa [2, 3, 6, 7, 10, 13, 14].

These new parasites were found on the gills of *Synodontis nigrita*, *S. velifer*, and *S. ocellifer* obtained from the Bagoué River. This type of parasitism, with three or more conspecific species of monogeneans parasites on a host species, is well known in Siluriformes. Indeed, N’Douba et al. [8, 9] described in Côte d’Ivoire different species of monogeneans of the genera *Quadriacanthus* and *Schilbetrema* from *Heterobranchus longifilis*, *H. isopterus*, and *Schilbe mandibularis*. Similarly, Mbondo et al. [7] described *Sy. melanoptera*, *Sy. apertipenis*, and *Sy. sanagaensis* on the gills of *S. rebeli* from Cameroon.

In this study, we also observed *Sy. melanoptera* on *S. nigrita* and *S. velifer*, but not on *S. ocellifer*. This monogenean has already been reported in Benin from *S. melanopterus* and in Cameroon from both *S. obesus* and *S. rebeli* [2, 7]. Accordingly, we can assume that *Sy. melanoptera* has a large geographical distribution in Africa given that it has been described from Benin, Cameroon, and Côte d’Ivoire. Likewise, this species, as is true with the other *Synodontella* spp., seems to have a wide host range including *S. melanoptera*, *S. obesus*, *S. rebeli*, *S. nigrita*, and *S. velifer*, and is thus stenoxenous [1]. Although *S. nigrita*, *S. velifer*, and *S. ocellifer* live in sympatry in the Bagoué river (at Samorossoba, Kanakono, Samorosso, N’Dara, and Guinguereni), we did not observe *Sy. melanoptera* from *S. ocellifer* even though, according to Paugy and Roberts [11], *S. ocellifer* is closely related to *S. velifer* and *S. nigrita*. The absence of *Sy. melanoptera* from *S. ocellifer* could be the result of a low sampling effort but can also be explained by an encounter problem in the compatibility filter [4].
